# Transforaminal Endoscopic Lumbar Discectomy for Migrated Disc Prolapse: A Retrospective Study of 169 Patients

**DOI:** 10.7759/cureus.99006

**Published:** 2025-12-11

**Authors:** Ajay Krishnan, Sandesh Subhash Agarawal, Bharat R Dave, Mahesh Sagar, Shivanand C Mayi, Ravi Ranjan Rai, Mirant B Dave, Mikeson Panthackel, Amritesh Singh, Arjit Vashishtha, Saurabh S Kulkarni, Yogenkumar Adodariya

**Affiliations:** 1 Spine Surgery, Stavya Spine Hospital and Research Institute, Ahmedabad, IND

**Keywords:** awake spine surgery, day-care surgery, foraminoplasty, lumbar disc herniation, migrated disc prolapse, minimally invasive spine surgery, percutaneous, transforaminal endoscopic lumbar discectomy

## Abstract

Background

The effectiveness of transforaminal endoscopic lumbar discectomy (TELD) has been questioned, but it is evolving. Although it provides acceptable, comparable, or even better results in sporadic reporting, TELD was contraindicated by many for migrated disc prolapse (MDP) and central disc prolapse (DP). This study aimed to evaluate the effectiveness of transforaminal endoscopic discectomy in MDP.

Methodology

The study was conducted at Stavya Spine Hospital and Research Institute between October 2010 and December 2023. All patients provided informed consent and underwent the procedure under local anesthesia. The inclusion criteria were lumbar degenerative DP with unilateral or bilateral symptoms at the L1-L5/S1 disc levels. Foraminal, extraforaminal, pure soft disc bulge, protrusion, extrusion, and calcified disc without craniocaudal migration were excluded. Intracanalicular-only extrusion and sequestration were included. Inside-out (IO) TELD was performed for all possible cases of low-migrated and high-migrated LDH. In cases of highly migrated LDH, the outside-out (OO) technique or transpedicular (TP) technique was also employed. If required, endoscopically visualized burr-foraminoplasty (FP) was added to the IO or OO primary techniques. The supra-facetal (SF) technique was employed when the highly migrated fragment was large, singular, and afresh (less than three weeks’ history). Carl Storz’s endoscopic system, Richard Wolf’s, Maxmore’s, or Joimax’s systems were used, along with self-designed instruments. Adequacy of decompression was confirmed by probing, fluttering dura, pulsatile dura, dural root fall-back, and MRI confirmation. Functional outcomes were assessed using the Numerical Rating Scale (NRS), Patient Satisfaction Index, return to basic work/job, and Oswestry Disability Index (ODI) scores.

Results

There were 169 patients with a minimum follow-up period of 24 months. Statistically significant (p < 0.05) ODI and NRS improvements were noted in all patients immediately and at six weeks without perioperative complications. All patients underwent daycare surgery and were able to resume their basic job activities within three weeks. Low, high, and very-high-migrated DP were 76, 62, and 31, respectively. OO was performed in 29 patients. Three patients underwent the TP approach, and 20 underwent the SF approach without foraminoplasty. The remaining patients underwent the IO technique (n = 117). Endoscopic FP was required in 27 cases of L5-S1 and at nine other spinal levels. Postoperative MRI showed adequate optimal decompression in all cases. The overall number of minor complications that had no long-term consequences was 18 (10.65%). Nine patients had residual fragments without any symptoms. Two patients with OO had a postoperative dysesthesia (POD) of the exiting nerve root. Five patients had protracted recovery of leg symptoms (POD traversing root) that took three weeks to resolve despite optimal decompression. No reoperations were required for MDP.

Conclusions

TELD decompression is an effective alternative for managing MDP. Excellent outcomes are possible even in highly migrated MDP, and TELD is no longer a contraindication.

## Introduction

Migrated disc prolapse (MDP) denotes the displacement of herniated disc material that has become dissociated from the originating intervertebral disc [1]. This migration may occur in both vertical and horizontal planes, with horizontal migration further categorized into central, paracentral, subarticular, foraminal, and extraforaminal types, and vertical migration subdivided into upward and downward displacements [2]. First described by Mixter and Barr in 1934, open discectomy involves performing a laminectomy or hemilaminectomy via a posterior midline approach, the transdural way [3]. By 1978, with sequential improvements, micro-lumbar discectomy (MLD) emerged as a less invasive, popular alternative to conventional open discectomy [4-6].

The management of migrated disc herniations poses specific challenges, particularly due to the fragmentation of disc material and its displacement beyond the standard interlaminar window [7]. Additionally, anatomical constraints, including pedicular obstruction and narrow foramina, limit intraoperative visualization, contribute to incomplete fragment excision, and increase the rates of surgical failure [8,9]. To overcome these limitations, refinements to MLD, such as fenestration techniques and translaminar approaches, have been developed to improve access to and visualization of migrated fragments [10].

The anatomical framework of Kambin’s triangle, introduced in the 1980s, has provided the foundation for transforaminal endoscopic lumbar discectomy (TELD) [11]. Subsequently, TELD, a fully endoscopic, minimally invasive technique, has gained traction for the treatment of lumbar disc prolapse [12-14]. Compared with MLD, TELD offers advantages such as reduced soft-tissue disruption, preservation of bony anatomy, and avoidance of dural or nerve root retraction [15,16]. However, early clinical experiences suggested that TELD was less effective in managing high-grade MDP, particularly at the L5-S1 level, due to restricted foraminal dimensions and complex pedicle anatomy, which hindered endoscopic access and visualization [17-19]. Consequently, MLD remained the preferred modality for addressing migrated disc pathology in such scenarios.

The introduction of foraminoplasty in TELD has substantially enhanced endoscopic access and visualization in anatomically challenging cases, broadening its applicability and efficacy in managing MDP [20]. Recent literature reports favorable outcomes for TELD in the treatment of high-grade MDP attributed to continuous improvements in surgical techniques, instrumentation, and procedural expertise. This study presents a retrospective analysis of TELD outcomes in patients with MDP with an emphasis on the technical nuances and a comprehensive review of current evidence.

This series was partly presented at KSSS 2021, Seoul; MISSABCON 2023, Pune; MISSABCON 2024, Gandhinagar; ERAS 2024, Portugal; MISSABCON 2025, Mumbai; and ASSICON 25, Bengaluru.

## Materials and methods

This retrospective study was conducted at Stavya Spine Hospital and Research Institute (SSHRI) between October 2010 and December 2023. The study protocol was approved by the Institutional Ethics Committee of SSHRI (protocol code: SSHRI/CS/NS/Migrated disc endo/AK/77/04.25) and the Clinical Trials Registry - India (CTRI/2025/04/085555). A total of 169 skeletally mature patients were enrolled in the study. Informed consent was obtained from all participants, who were counseled regarding potential conversion to open surgery if optimal decompression could not be achieved endoscopically. Patients were offered various surgical options, including MLD, spinal fusion, and TELD. Those who opted for TELD and fulfilled the inclusion criteria were included in the analysis.

Inclusion criteria comprised patients with lumbar disc prolapse (DP) characterized by vertically migrated (craniocaudal) intracanalicular disc extrusion or sequestration involving lumbar levels from L1-L2 to L5-S1, with acute-onset symptoms unresponsive to adequate conservative management and a minimum postoperative follow-up of 24 months. Patients were excluded if they had foraminal or extraforaminal disc herniations, non-migrated soft disc bulges or protrusions, or calcified disc herniations without craniocaudal displacement. Further exclusions included spinal pathologies such as congenital canal stenosis, cauda equina syndrome, multilevel degenerative stenosis, instability, tumors, infections, inflammatory disorders, or less than 24 months of follow-up. These criteria ensured a homogeneous study population for accurate assessment of TELD outcomes in vertically migrated lumbar disc herniations.

Demographic details, including age, sex, body mass index (BMI), and comorbidities, were recorded. Clinical details included duration of symptoms, preoperative and postoperative pain assessments using the Numerical Rating Scale (NRS) for both back and leg pain, neurological status based on Medical Research Council (MRC) grading (with grade <3 considered a significant deficit), and functional disability evaluated using the Oswestry Disability Index (ODI) [21] and NRS [22]. The ODI is a validated, self-administered questionnaire used to quantify disability due to lower back pain. It consists of 10 sections, each scored from 0 to 5. The total score is calculated as follows: (sum of section scores ÷ 50) × 100, yielding a percentage score. The results are interpreted as follows: 0-20%, minimal disability; 21-40%, moderate disability; 41-60%, severe disability; 61-80%, crippled; and 81-100%, bed-bound or symptom exaggeration. The NRS is a validated, patient-reported scale used to quantify pain intensity on a 0-10 continuum from 0 (no pain) to 10 (worst imaginable pain), enabling robust comparison across clinical time points.

Surgical technique

All patients undergoing TELD were admitted on the day preceding surgery. Preoperatively, each patient received alprazolam 0.25 mg and pregabalin 75 mg orally the night before the procedure to facilitate anxiolysis and analgesia. On the day of surgery, conscious sedation was administered in conjunction with local anesthesia. One hour before the procedure, intramuscular midazolam (0.05 mg/kg) and diclofenac sodium (75 mg) were administered. A bolus of intravenous fentanyl (1 mg/kg) was given approximately 10 minutes before the skin incision, with additional doses titrated intraoperatively based on patient feedback. An anesthesiologist was present throughout the surgical procedure to monitor sedation and vitals. TELD was performed via Kambin’s triangle using fluoroscopy guidance. The skin entry point was determined by the level of DP-specific technique employed, typically 7-15 cm lateral to the midline, at an angulation of 15°-20° in the axial plane. Higher obliquity in the outside-out (OO) approach and the highest obliquity in the supra-facetal (SF) approach were considered, respectively. For higher lumbar levels, a more medial entry point was utilized. Local infiltration was achieved using a 1:1 mixture of 1% lidocaine and 0.5% bupivacaine.

One of four established TELD techniques was employed depending on the morphology and migration of the disc herniation: the inside-out (IO), OO (blind reamed foraminoplasty or endoscopically visualized burred foraminoplasty), SF, or transpedicular (TP) technique.

The IO technique was the primary approach in most cases involving low- and high-grade MDP, particularly when the herniated fragment remained attached or was traceable near the parent disc. In this technique, the needle was inserted approximately 10-15 cm from the midline, at a level a few centimeters above or below the involved disc, with initial contact made at the facet joint. The needle was advanced ventrally over the facet to reach the disc, with its tip ultimately positioned at the medial pedicular line on anteroposterior fluoroscopy and the posterior vertebral body line on lateral views. Needle trajectory was fine-tuned using the bevel orientation and craniocaudal or dorsoventral adjustment of the stylus hub. Approximately 1.5 mL of the anaesthetic mixture was administered before the annular puncture. A guidewire was then passed through the needle, over which a tapered dilating trocar was inserted. Once the trocar contacted the posterolateral disc margin, it was repositioned for optimal approach, followed by additional local infiltration and malleting into the disc. The bevelled working cannula was then advanced over the trocar, and the endoscope was introduced (Maxmore System, Richard Wolf, Karl Storz Gore System, Germany).

A full-endoscopic spine surgery uniportal system with continuous saline irrigation was used to maintain a clear operative field and achieve hemostasis by sustaining irrigation pressure between 30 and 50 mmHg. Saline (0.9%) was administered via gravity flow and free drainage, with an intermittent manual pump utilized when excessive bleeding or obscuring epidural fat was encountered.

Under direct endoscopic visualization, subannular free fragments were removed, and when necessary, the annulus fibrosus and posterior longitudinal ligament were incised for complete fragment retrieval. “Joystick” manipulations, namely, medialization, dorsalization, rostralization, caudalization, and horizontalization, of the endoscope working sheath assembly were performed to navigate the intracanal space and access the migrated fragments.

The SF technique was reserved for significant, singular, and recent (less than three weeks old) high-grade migrated disc fragments. For completely sequestrated, high- or very-high-migrated disc prolapse, the OI technique or the TP approach was used to access the epidural space. In such cases, endoscopically visualized burr foraminoplasty and dorsal corporeal burring were also employed to enhance visualization and facilitate the retrieval of distant or obstructed fragments.

Technique selection was individualized based on preoperative planning, taking into account factors such as the extent of disc migration, facet morphology (e.g., enlarged or squared facets), and the specific level involved (e.g., L5-S1). Endoscopic burr foraminoplasty was incorporated into any of the four approaches when required to improve working space and visibility. Once optimal positioning of the endoscope and working sheath was achieved, disc fragments were removed either en masse or piecemeal under continuous visualization. Straight graspers were used when the fragment aligned with the endoscopic axis, while curved or bendable instruments such as articulating hooks and flexible graspers were employed when off-axis removal was needed. Manipulative techniques, including pulling, wriggling, and twisting, facilitated the extraction of adherent fragments. Complete removal of the offending migrated disc was confirmed intraoperatively using standardized endoscopic endpoint criteria, as previously described by the authors [12]. Postoperative adequacy of decompression was assessed by MRI within three hours of surgery and interpreted by a musculoskeletal radiologist. A single absorbable suture was placed at the incision site. Additional intraoperative data, including operative time, fluoroscopic exposure (shot count), and any intraoperative events, were documented. Both major and minor perioperative and postoperative complications were systematically recorded.

Postoperative protocol

The patient was made to walk on the same day and discharged if they were comfortable. The length of hospital stay was assessed in relation to clinical outcomes, including the number of days until resumption of basic work, NRS, ODI, and Patient Satisfaction Index, which were noted during subsequent visits. A response of 1 or 2 was considered a satisfying outcome, and 3 indicated a dissatisfied outcome, as per the Patient Satisfaction Index [23]. The neurological recovery rate was evaluated as the percentage of patients with preoperative neurological deficits (less than MRC grade 3 in any myotome) that improved to MRC grade 3 or more.

Statistical analysis

Statistical analysis was performed using SPSS version 20.0 (IBM Corp., Armonk, NY, USA). Continuous variables were expressed as mean ± standard deviation (SD), and categorical variables as frequencies and percentages. Pre- and postoperative NRS and ODI scores were compared using the paired t-test. Between-group comparisons of clinical and demographic variables were analysed using independent t-tests. Subgroup comparisons based on disc migration grades were conducted using one-way analysis of variance. A p-value <0.05 was considered statistically significant.

## Results

The study involved 169 patients, with a notable male predominance: 65.1% (n = 110) were male, while 34.9% (n = 59) were female. The participants had a mean age of 40.7 ± 10.3 (20-64) years. Comorbidities were present in a subset of patients: hypertension in 28 (16.9%) patients, diabetes mellitus in eight (4.8%), coronary artery disease in nine (5.4%), asthma in two (1.2%), and chronic corticosteroid use in three (1.8%) patients. A history of previous spinal surgery was noted in eight (4.8%) patients. All patients had undergone an adequate trial of conservative management, with a mean duration of 5.6 ± 2.3 weeks (range = 1-13 weeks). Preoperative motor power, as assessed by the MRC scale, averaged 3.9 ± 1.01 (range = 1-5), as depicted in Table 1.

**Table 1:** Baseline demographic and clinical profile of patients undergoing surgery (n = 169).

Variable	Mean ± SD, frequency (%) (n = 169)
Age (years)	40.7 ± 10.3 (range = 20–64)
Body mass index (kg/m²)	28.4 ± 3.9 (range = 19.5–39.3)
Gender	
Male	110 (65.1%)
Female	59 (34.9%)
Comorbidities	
Hypertension	28 (16.9%)
Asthma	2 (1.2%)
Diabetes mellitus	8 (4.8%)
Chronic steroid use	3 (1.8%)
Coronary artery disease	9 (5.4%)
History of previous spine surgery	8 (4.8%)
Duration of conservative treatment (weeks)	5.6 ± 2.3 (1–13)
Preoperative MRC power	3.9 ± 1.01 (1–5)

**Figure 1:**
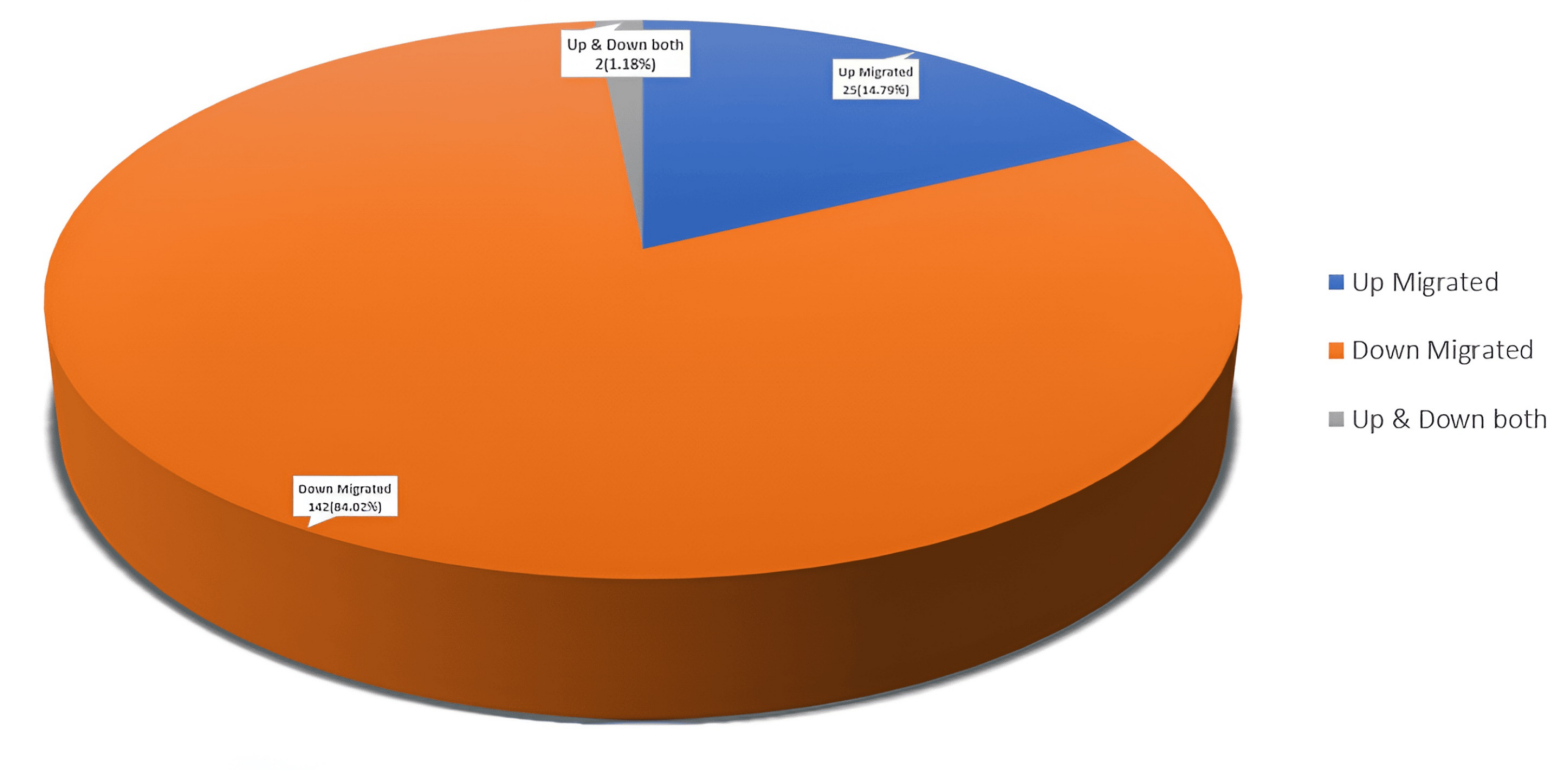
Direction of disc migration.

Cohort analysis (n = 169) revealed that 44.9% (n = 76) of patients exhibited low-grade MDP, 36.6% (n = 62) presented with high-grade migration, and 18.3% (n = 31) demonstrated very high-grade migration, based on the modified Lee grading criteria. Downward (caudal) migration was observed in 84.02% (n = 142) of cases, while upward (cranial) migration accounted for 14.79% (n = 25). Two patients presented with both upward and downward migration patterns, as described in Figure 1. Classification according to the Michigan State University system identified type 2AB herniation as the most common subtype (64, 37.86%), followed by type 3AB (43, 25.44%) and type 2A (36, 21.30%).

**Figure 2:**
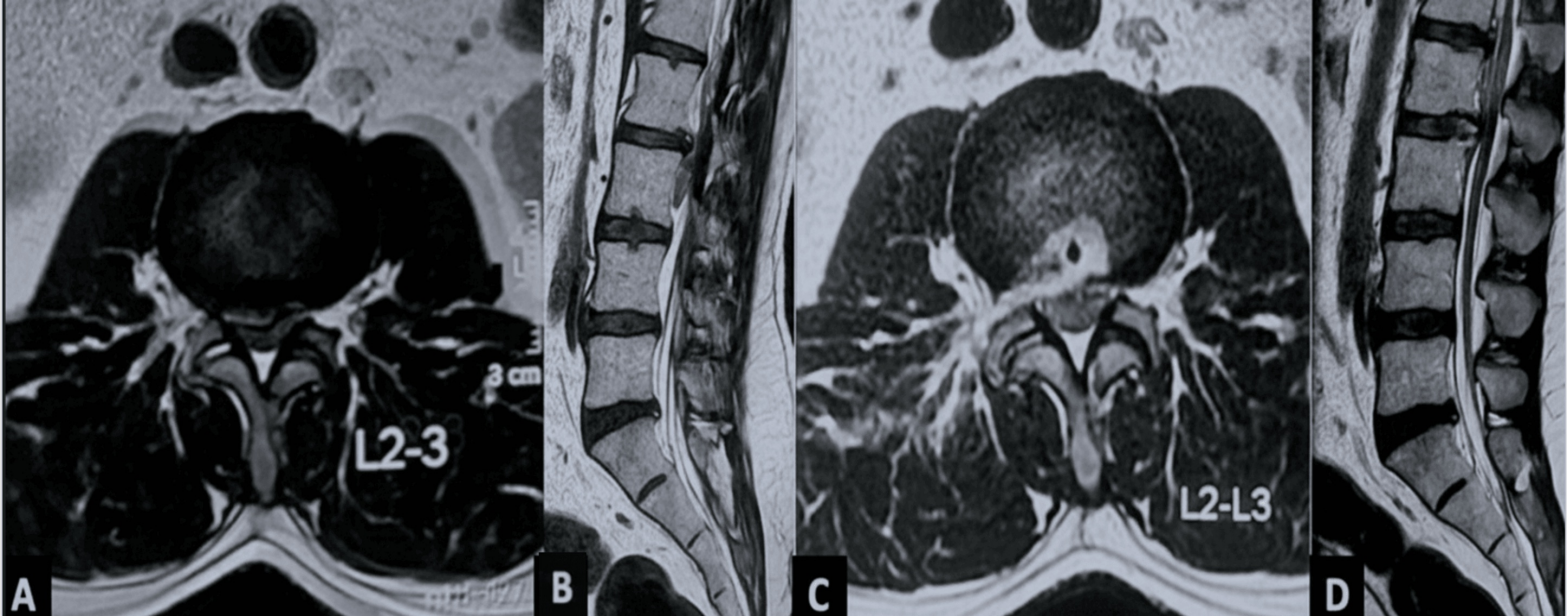
(A, B) Preoperative MRI of a 35-year-old male demonstrating a high-grade downmigrated right-sided disc prolapse reaching the L3 pedicle, classified as Michigan State University type 2AB. (C, D) Postoperative MRI confirming complete fragment removal following the inside-out technique without foraminoplasty.

**Figure 3:**
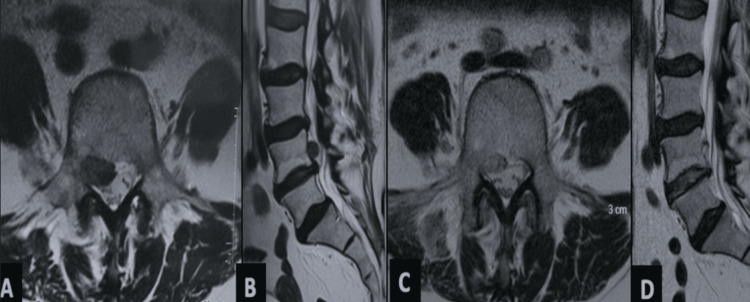
(A, B) Preoperative MRI of a 58-year-old male showing a high upmigrated right-sided disc prolapse reaching the L4 pedicle, Michigan State University type 2AB. (C, D) Postoperative MRI following the inside-out technique under local anesthesia without foraminoplasty, demonstrating adequate decompression with a residual minute fragment and no surgical footprints. The patient had an uneventful, symptom-free follow-up.

**Figure 4:**
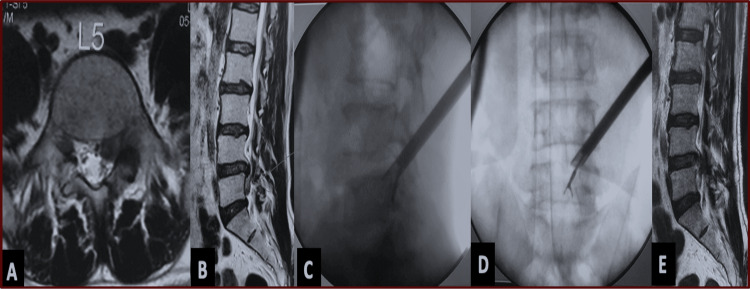
(A, B) Preoperative MRI of a 43-year-old male showing a very high-migrated left-sided disc reaching below the L5 pedicle, Michigan State University type 2AB. (C, D) Intraoperative image intensifier views during surgery performed under local anesthesia via the outside-in technique with endoscopic burred foraminoplasty. Note the highly oblique endoscopic trajectory on both anteroposterior and sagittal planes. (E) Postoperative sagittal MRI T2 image confirming fragment removal and decompression.

The mean operative time was 74.5 ± 13.2 minutes, with an average fluoroscopic shoot count of 18.6 ± 4.5 per procedure. The majority of cases (69.2%, n = 117) were performed using the IO technique, as depicted in Figure 2, followed by the OI technique in 17.2% (n = 29), as depicted in Figure 3, the SF technique in 11.8% (n = 20), and the TP technique in 1.8% (n = 3). Blind foraminoplasty was performed in 28.4% (n = 48) of patients, while visualized burr foraminoplasty, as depicted in Figure 4, was performed in 0.5% (n = 1). The mean final follow-up duration was 131.2 ± 121.8 months, ranging from 10 to 500 months. The Patient Satisfaction Index averaged 1.1 ± 0.3. The mean time to return to basic work or job activities was 19.5 ± 3.7 days (range = 6-31 days), as depicted in Table 2.

**Table 2:** Intraoperative parameters and postoperative recovery outcomes.

Variable	Mean ± SD/Frequency (%)
Operative time (minutes)	74.5 ± 13.2
Fluoroscopic shoots	18.6 ± 4.5
Basic technique	n = 169
Inside-out	117 (69.2%)
Supra-facetal	20 (11.8%)
Outside-out	29 (17.2%)
Transpedicular	3 (1.8%)
Foraminoplasty	n = 59
Blind reamed foraminoplasty	11 (18.6%)
Endoscopic visualized burred foraminoplasty	48 (81.4%)
Final follow-up (months)	131.2 ± 121.8 (range = 10–500)
Patient Satisfaction Index	1.1 ± 0.3
Return to basic work/job (days)	19.5 ± 3.7 (range = 6–31)

There was a statistically significant difference between ODI and NRS scores of leg and back pain from preoperative to the final follow up visit, as depicted in Table 3.

**Table 3:** Time-based comparison of functional and pain scores using ANOVA and paired t-tests. Changes in Oswestry Disability Index (ODI) and Numerical Rating Scale (NRS) for back and leg pain over time. ODI values across multiple time points were compared using repeated-measures analysis of variance (ANOVA) (F-statistic reported). NRS scores, assessed at two time points (preoperative and postoperative), were compared using paired t-tests (t-statistic reported). Values are presented as mean ± standard deviation. Sample size is 169 at all time points.

Variable	Time point	Mean ± SD	Statistics	P-value
ODI	Preoperative	80.7 ± 13.1	ANOVA	<0.05
	Third-month follow-up	17.8 ± 4.5		
	Sixth-month follow-up	10.2 ± 3.7		
	24th-month follow-up	5.9 ± 3.04		
	Final follow-up	6.06 ± 3.5		
NRS (back pain)	Preoperative	5.2 ± 2.5	(paired t-test)	<0.05
	Postoperative	1.6 ± 0.54		
NRS (leg pain)	Preoperative	8.5 ± 1.4	(paired t-test)	<0.05
	Postoperative	0.75±0.79		

The follow-up was 131.2 ± 121.8 (range = 10-500) months. Eight patients were lost to follow-up during the latest follow-up, despite the results being available and included in the two-year follow-up. During the surgery, it was observed that one case developed a chip facet fracture (no additional management needed), while the other two experienced a prodrome of convulsion. Following surgery, all but three of the 15 patients with an MRC power of less than 3 recovered within six weeks. A motor recovery rate of 80% was noted.

**Figure 5:**
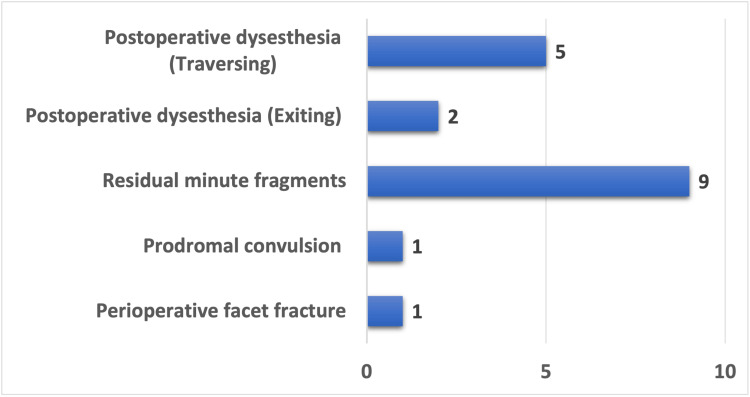
Intraoperative and postoperative complications. Intraoperative complications included perioperative facet fracture and prodromal convulsion. Postoperative complications included residual minute fragments, postoperative dysesthesia (exiting), and postoperative dysesthesia (traversing). A total of 18 (10.65%) such complications were documented.

Additionally, nine cases experienced complications in the form of residual fragments that were not symptomatic (Figure 5). Seven patients suffered from postoperative dysesthesia (exiting root; n = 2, traversing root; n = 5). Conservative management resolved the dysesthesia in all patients within six weeks. Overall, 18 (10.65%) minor complications were identified, each resolving spontaneously without any enduring adverse outcomes.

## Discussion

Lombardi reported the earliest documented case of MDP in 1973 [24]. In recent years, full-endoscopic spine surgery has become a leading ultra-minimally invasive technique for treating lumbar DP. Among the available surgical approaches, such as interlaminar and TELD, comparative studies remain limited [13]. Initially, TELD was contraindicated for high-grade MDP, but its use has expanded to include even very high-grade migrations. Compared to the interlaminar approach, TELD offers several advantages, including a lower risk of dural sac injury, preservation of posterior anatomy, direct access to the disc, the ability to perform under local anesthesia, and avoidance of neural retraction.

In this study, the mean patient age was 40.7 ± 10.3 years, with the majority of patients aged 41-60 years. Males comprised 65.1% (n = 110), and females 34.9% (n = 59). The L4-L5 segment was most commonly affected (61.2%), consistent with prior literature [25,26]. While disc herniations can be classified by axial location [7], we used the Michigan State University classification for its objective assessment of disc size and canal occupancy. Most patients had significant canal compromise, classified as Michigan State University grades 2 or 3 (occupancy exceeding 50%). Migration classification followed Lee et al.’s system, later refined by Kim et al. [27], and was subsequently consolidated into low-, high-, and very-high-grade categories in the recent literature [28-30], which we adopted here. To our knowledge, this represents the most extensive series of MDP treated with TELD [7,8,12,13], including the highest number (n = 31) of very-high-grade migrated cases reported [7,13,14,19].

Four different TELD techniques were employed based on the extent and direction of migration. The IO technique was the most common (117 patients, 69.2%; Figures 2, 3), being suitable for both low- and high-grade migrations. After releasing annular anchorage, the bevelled sheath is carefully withdrawn, angled cranially or caudally, and advanced across the disc into the epidural space. The OO technique with foraminoplasty was performed in 29 (17.2%) patients, primarily for high- and very-high-grade downward migrations (Figure 4), as described in prior studies [7,31-33]. The SF approach, used in 20 (11.80%) patients, provides direct epidural access without foraminoplasty and employs a more medial entry than OO. Both OO and SF bypass the disc space to reach the epidural compartment directly.

Most IO and foraminoplasty variations aim to enlarge the inferior foramen, ventromedial superior articular process, pedicle, or posterosuperior vertebral body to align with the migrated fragment for effective removal [23,31-33]. Foraminoplasty tools include mills, trephines, reamers, burrs, articulated burrs, and lasers supplemented by flexible instruments to extend reach beyond the endoscopic view. Newer transforaminal modifications for highly migrated herniations include TP, contralateral transforaminal, and double-level approaches [34-37]. In our cohort, the TP approach was used in three patients with fully sequestrated, highly migrated fragments.

Postoperative dysesthesia often results from mechanical irritation, improper technique, or thermal injury from radiofrequency ablation, which can impact recovery and quality of life [37]. Seven patients developed POD; only two had new exiting nerve root dysesthesia, both after blind reamed foraminoplasty. No POD occurred with visually guided endoscopic burred foraminoplasty, supporting its preferential use to minimize nerve irritation. Intraoperative seizures during TELD are rare but may relate to elevated epidural pressure [21,38]. Two patients exhibited prodromal seizure-like symptoms managed by pausing the procedure for 15 minutes and head end elevation. One minor intraoperative facet fracture occurred without clinical consequence. Residual disc fragments identified in nine asymptomatic cases underscore the importance of preoperative imaging to assess fragment integrity, as fragmented discs require more extensive removal.

Fluoroscopy is indispensable in minimally invasive spine surgery but raises concerns about radiation exposure [39-41]. Foraminoplasty requires repeated fluoroscopic guidance to protect the exiting nerve root, with reported shot counts ranging from 26 to 40 [42,43]. Yang et al. described endoscopically visualized burr foraminoplasty with a mean fluoroscopy time of 26 seconds in elderly patients [44]. This procedure primarily utilizes fluoroscopy to position the sheath, performing foraminoplasty with visualization rather than relying on blind fluoroscopic guidance [45].

Cai et al. reported fewer fluoroscopic shots (6.03 ± 1.91) during foraminoplasty for highly down-migrated herniations, compared to 18.6 ± 4.5 during conventional TELD in our study [20]. Chen et al. described a supra-pedicular extracorporeal approach requiring 10.5 ± 1.8 shots [46]. Although our fluoroscopy usage was relatively high, Ahn et al. estimated that, with lead apron protection, over 5,000 procedures could be safely performed annually, compared with only 291 without protection. Ultrasound-guided or navigation-assisted TELD may further reduce radiation exposure [47].

Operative durations vary by technique. Cai et al. reported 92.7 ± 24.1 minutes for fully endoscopic foraminoplasty in highly down-migrated cases. Chen et al. [21] reported 65.5 ± 7.1 minutes for the supra-pedicular extracorporeal technique. Kim et al. noted a 90-minute supra-pedicular circumferential opening during TELD for high-grade inferior migration. Qiao et al. [48] found that the trans-SAP approach significantly reduced operative time (63.2 ± 11.3 minutes) versus conventional TELD (78.4 ± 18.3 minutes), suggesting greater efficiency. Jiang et al. reported 67.7 ± 12.5 minutes for the TP transforaminal approach [49]. Our mean operative time of 74.5 ± 13.2 minutes is consistent with these findings.

Significant functional improvements were observed, with immediate postoperative reductions in NRS for back and leg pain and sustained ODI decreases at long-term follow-up (131.2 ± 121.8 months). These outcomes align with existing literature on TELD for MDP. The Patient Satisfaction Index averaged 1.1 ± 0.3, indicating high satisfaction. Hospital stays were typically daycare, with a return to work or activities averaging 19.5 ± 3.7 days. An 80% motor recovery rate was achieved.

TELD requires substantial surgical training to develop the requisite skills, which are often lacking in standard residency or fellowship curricula. Gadjradj et al. [50] demonstrated TELD’s cost-effectiveness and non-inferiority for DP in a multicenter randomized trial. Their results, as well as those of others, suggest that endoscopic discectomy is cost-effective, practical, and teachable with proper mentorship [51]. Because TELD has traditionally been contraindicated for MDP, conventional open surgery should be preferred during the early learning curve, with a low threshold for conversion in early TELD cases if needed. Although our learning phase yielded favorable outcomes, treatment decisions must prioritize surgeon expertise and comfort. This study presents the most extensive series (n = 169), including 31 very-high-grade migrations, detailing surgical techniques with effective clinical outcomes.

Limitations include the retrospective, non-randomized design, lack of a control group, and non-continuous sampling. Results may not generalize to settings that use alternative endoscopic, minimally invasive, or open surgical procedures. Despite using the latest classification system for migration grade and direction, variability persists due to differences in spinal level, age, and anatomy. All L5-S1 cases via the supra-iliac transforaminal approach were included; cases using trans-iliac approaches for complex migration patterns were excluded. Calcified discs or concurrent stenosis cases were excluded to reduce heterogeneity. Although we categorized surgical approaches into four types, complete standardization remains a challenge. The predominance of IO reflects the senior author’s early preference, with other techniques introduced later, potentially biasing the results. Finally, the procedure cost was higher than microdiscectomy or fusion, potentially affecting patient selection and perceived value.

## Conclusions

TELD has emerged as a highly effective and minimally invasive surgical approach for managing MDP. This technique has proven to deliver favorable outcomes, even in cases involving significant or severely migrated discs. Its success underscores the method’s growing importance as a preferred intervention in the surgical treatment of migrated disc herniation. By minimizing tissue disruption and promoting faster healing, it not only offers patients quicker recovery times but also reduces postoperative pain. Additionally, the procedure has been shown to significantly enhance patient satisfaction, resulting in a higher quality of life following surgery.
